# Prevalence, motivation, and associated factors of medicinal herbs consumption in pregnant women from Eastern Mediterranean Regional Office: a systematic review

**DOI:** 10.1080/13880209.2023.2229388

**Published:** 2023-07-14

**Authors:** Afaf Bouqoufi, Laila Lahlou, Fatima Ait El Hadj, Mohammed Abdessadek, Majdouline Obtel, Youssef Khabbal

**Affiliations:** a Laboratory of Innovation Research in Health Sciences, Therapeutic Innovation, Translational Research, and Epidemiology, Ibn Zohr University, Agadir, Morocco; b Laayoune Higher School of Technology, Ibn Zohr University, Laayoune, Morocco; c Social Medicine, Public Health, Hygiene and Preventive Medicine Laboratory, Department of Public Health, Mohammed V University, Rabat, Morocco

**Keywords:** Frequency, herbal medicine, phytotherapy, plants consumption, pregnancy

## Abstract

**Context:**

Worldwide access to medication remains a major public health problem that forces pregnant women to self-medicate with several sources, such as medicinal plants. This alternative medicine is increasing in many low- and high-income countries for several reasons.

**Objective:**

This a systematic literature review on the prevalence of herbal use during pregnancy from the World Health Organization (WHO) Eastern Mediterranean Regional Office.

**Methods:**

Cross-sectional studies were searched from January 2011 to June 2021 on PubMed, Scopus, and Web of Science. We used the Rayyan website to identify the relevant studies by screening the abstracts and titles. These were followed by reading the full texts to identify the final studies to be included. The data were extracted, and the quality of the studies was assessed using the quality appraisal tool.

**Results:**

Of the 33 studies included in this review, 19 were conducted in Iran, 5 in Saudi Arabia, 4 in Palestine, 2 in Egypt, and 1 each in Oman, Iraq, and Jordan; the prevalence of herbal medicine use among pregnant women varied from 19.2% to 90.2%. Several plants were mentioned for pain management during the pregnancy period. The findings suggest family and friends are major motivating sources for the use of herbal medicine.

**Conclusions:**

The wide variety of herbal products used in this study reflects the traditions and geographic diversity of the region. Despite the importance of literature-based data about the use of herbal medicine, it is necessary to obtain knowledge, attitude, and motivation for herbal consumption among pregnant women.

## Introduction

Herbal medicines include herbs, herbal materials, herbal preparations, and finished herbal products, that contain active ingredients that are parts of plants, other plant materials, or combinations of these (World Health Organization [WHO], 2019). Herbal treatment is based on the extract of the whole plant, part of the plant (i.e., leaves, roots, flowers), or a mixture of several herbal compounds. For several years, herbal remedies have been taken as a preventive measure to maintain health and to prevent, relieve, or cure diseases (Pieroni et al. 2005). Approximately 80% of the world’s population uses various traditional medicines, including herbal medicines, to diagnose, prevent, and treat disease, and to improve general well-being (Eisenberg et al. 1998). This practice is due to the popular belief that herbs are natural and free of any adverse effects compared to conventional medicine (Pieroni et al. 2005). Local traditions and social pressure, for example, high costs of drugs and medical visits, as well as insufficient health coverage, could also be the reason behind this practice (Choudhry 1997). Herbal medicines are available as non-prescription medicines. Given such ease of access, most women say that they decided to use herbal medicine on their own initiative or on the advice of family and/or friends (Kennedy et al. 2016).

In Eastern Mediterranean countries, especially in the Arab world, traditional medicine has always been practised despite the advances in modern medicine. The herbalists and scientific community have become more interested in the concept of traditional Arabic herbal medicine (Azaizeh et al. 2010). For example, an exhaustive study including 63 articles in total on Ethnobotanico-pharmacological studies was carried out in Morocco, from 1991 to 2015 (Fakchich and Elachouri 2021). Another example of a research study on this subject was conducted with a specific focus on pregnant women, This work was the subject of a systematic review of herbal medicine use during pregnancy in Sub-Saharan Africa (El Hajj and Holst 2020). The first trimester of pregnancy is associated with physiological changes including nausea, vomiting, constipation, and gastric problems. In the third trimester, gastroesophageal reflux and uterine contractions are more common. Pregnant women self-medicate by using herbs or herbal remedies to relieve the sympathetic signs of pregnancy (John and Shantakumari 2015). For this reason, women have been identified as the major users of medicinal herb products. Its prevalence is up to 60% in developed countries (Hall et al. 2011). In addition, a literature search of numerous studies from the Western world reported that the prevalence of herbal medicine use in pregnancy ranged from 1 to 60%. Moreover, the prevalence rates were 58% in the UK (Holst et al. 2011), 48% in Italy (Lapi et al. 2010), 40% in Norway (Nordeng et al. 2011), 34% in Australia (Frawley et al. 2013), and 6–9% in the USA and Canada (Moussally et al. 2009; Louik et al. 2010). These different rates of prevalence depend on geographic location, ethnicity, socioeconomic status, and cultural traditions (Illamola et al. 2019). In this context, the use of herbs during pregnancy can have deleterious consequences for the mother and fetus. It poses a major challenge for healthcare because most patients are not informed about herbal uses (Bercaw et al. 2010).

The main aims of our systematic review were to retrieve primary literature reporting the prevalence of herbal medicines used in pregnancy and the postnatal period, as well as to investigate women’s experiences, motivations, and risk factors associated with the use of herbal medicines during pregnancy.

## Methods

The PRISMA checklist was used to guide the reporting of the systematic review. A systematic review protocol was registered by PROSPERO 2021 with ID: CRD42021264368.

### Eligibility criteria

Original articles in human studies that focused on pregnant or post-natal women based on the cross-sectional survey were considered eligible to be included in this review. Studies are also included if they describe the prevalence, attitudes, or beliefs of women towards herbal medicines or provide information about the use of herbs, and herbal products and therapies during pregnancy, including the type of herbal products, conditions of use, and source of information. We excluded unpublished reports, pilot studies, conference abstracts, opinion pieces, editorials, seminal works, and systematic reviews. Studies were excluded if they focused on women’s use of herbal medicines for other conditions that were not related to maternal health care. Studies that reported the combined use of herbal medicines and drugs were excluded if the data on herbal medicines could not be separated sufficiently, and we excluded animal research.

### Information sources and search strategy

Two authors, Afaf Bouqoufi and Laila Lahlou, performed independent searches on PubMed, Scopus, and Web of Science for articles published from January 2011 to 2021. The search was conducted using the Boolean operators AND OR which narrowed and widened the search and used a combination of MeSH (medical subjects heading). The following search string was used: “herbal medicine” OR plants OR «traditional medicine» OR herbs OR “herbal therapy” AND pregnancy* with a special focus on different countries from the Eastern Mediterranean Regional Office (EMRO). The regional office of the WHO serves 23 countries and territories in the Middle East, North Africa, the Horn of Africa, and Central Asia. including (Afghanistan, Bahrain, Djibouti, Egypt, Iran, Iraq, Israel, Jordan, Kuwait, Lebanon, Libya, Morocco, Algeria, Oman, Pakistan, Qatar, Saudi Arabia, Somalia, Sudan, Syria, Tunisia, the United Arab Emirates, Palestine, and Yemen) World Health Organization Eastern Mediterranean Regional Office [Internet] Switzerland (2021): WHO; [cited July 5th, 2021]. Available from: http://www.emro.who.int/countries.html

### Selection process

We used Rayyan (http://rayyan.qcri.org), a free web and mobile app, that helps expedite the initial screening of abstracts and titles using a process of semi-automation. This was followed by reading the full texts to identify the eligible studies. The references in each article were hand-searched for additional eligible studies. Finally, all articles were imported into Zotero, a bibliographic management software system.

### Data collection process and data items

Using eligibility criteria, we extracted data including country of studies, year of publication, participant’s demographics, the prevalence of herbal medicine use, details of herbal medicines used, characteristics of users, maternal conditions treated by herbal medicines, reasons for use, and source of information.

### Study risk of bias assessment

The quality of eligible studies was assessed. This process was conducted using the Joanna Briggs Institute (JBI) 2021 an international research organization based in the Faculty of Health and Medical Sciences at the University of Adelaide, South Australia. The purpose of this appraisal is to assess the methodological quality of a study and to determine the extent to which a study has addressed the possibility of bias in its design, conduct, and analysis.

**Figure 1.**
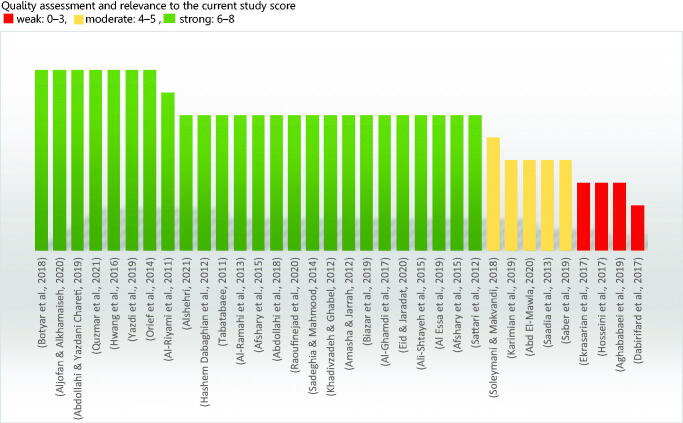
Result of quality appraisal using the JBI appraisal tool.

The Quality assessments were conducted by two independent authors (Afaf Bouqoufi and Laila Lahlou) using a recent version of the Joanna Briggs Institute’s critical appraisal tools Checklist for Analytical Cross Sectional Studies. Critical Appraisal Tools | Joanna Briggs Institute. Joanna Briggs Institute’s critical appraisal tools [Internet] | Australia: JBI; [cited 2021 August 31]. Available from: https://jbi.global/critical-appraisal-tools. A third author (Youssef Khabbal) was consulted if consensus could not be reached. When information is missing from the studies, we contacted the authors *via* email. All observational studies were included irrespective of quality score. The articles with missing data were included as long as they presented the prevalence of plant use. The findings of the quality appraisal of eligible studies were reported in Figure 1.

## Results

### Study selection

**Figure 2.**
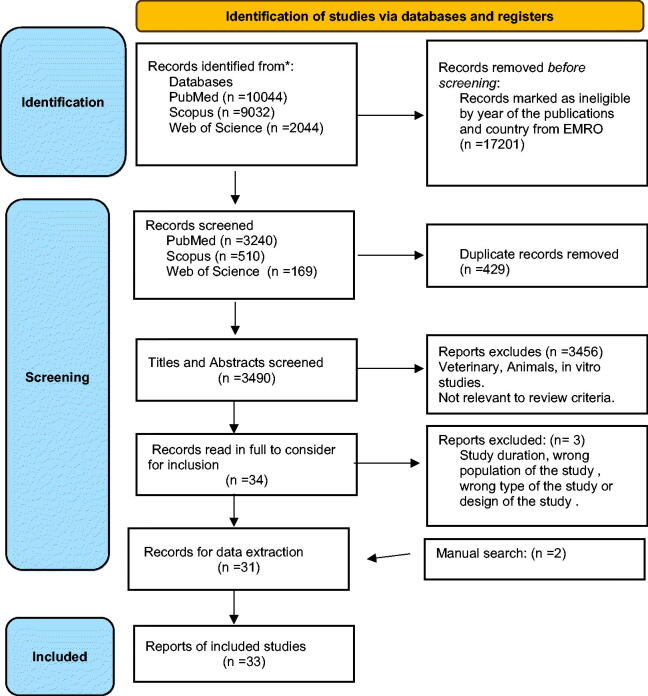
Systematic review flowchart.

The flowchart of the studies included in this systematic review is illustrated in Figure 2. The research generated 21,120 articles, of which 429 records were duplicates and were removed: 3459 others were excluded as ineligible after reading their titles or abstracts. Full texts of the remaining 34 records were downloaded and screened or in some cases, the full texts were screened online. After screening through the eligibility criteria, three studies were considered ineligible. A total of 31 studies were found eligible after we added two items in a simple way of research. Therefore, 33 studies were included in the systematic review.

### Study characteristics

This present review includes 33 papers in total, of which 19 were conducted in Iran, 5 in Saudi Arabia, 4 in Palestine, 2 in Egypt, and 1 each in Oman, Iraq, and Jordan (Table 1). The oldest studies in terms of year of publication (2011) are in Iran (Tableaee 2011) and Oman (Al-Riyami et al. 2011). The most recent one is in Palestine (Quzmar et al. 2021). All of the included studies used structured or semi-structured survey questionnaires to collect data among pregnant women on the use of herbal medicine during pregnancy, type of herbal products, condition of use, source of information, and referral source. One study from Iran (Khadivzadeh and Ghabel 2012) employed the largest sample size of 919 women, whereas the study from Oman (Al-Riyami et al. 2011) recruited only 139 participants. The prevalence of the use of herbal medicines among pregnant women in the Eastern Mediterranean Regional Office region varied from 19.2% (Soleymani and Makvandi 2018) to 90.2% (Dabirifard et al. 2017).

**Table 1.** User profile of studies on herbal medicine use among pregnant women from the EMRO region.

Nº	Study	Country	Sample Size	Study design	Mean age (Years)	Educational level (years of education)	Employement status	Residence	Partity (Mean pregnancy number)	Trimester
1	(Botyar et al. 2018)	Iran	210	This cross-sectional, descriptive, analytical study	>20 : 0	Illiterate : 1.45	Homemaker : 86.3 %	City : 93.2%	NR	NR
					21-30 : 53.4%	Primary school : 12.3 %	Practitioner : 13.7 %	Village : 6.8 %		
					31-40 : 42.5%	Middle school : 19.3%				
					41-45 : 4.1 %	Hight school : 49.3%				
						University : 17.8%				
2	(Saadia et al. 2013)	Saudi Arabia	360	a self-administered crosssectional survey.	Below 25. : 20.8 %	Primary or below 35.8 %	Housewife : 81.4 %	NR	Primiparous : 14.6 %	puerperium
					25–30 : 33.8 %	Middle school 31.8 %	Teaching : 13.2 %		G2–4 : 43.7 %	
					Above 30 : 45.45%	High school 20.3 %	Professional : 2.3%		G5 and above 1: 41.7%	
						College and above 12.1%	Government service : 3.1%			
3	(Biazar et al. 2019)	Iran	836	descriptive cross-sectional study	≤20 : 25.3%	Illiterate : 1.5 %	Employed : 3.8%	Urban : 72.3%	NR	NR
					20-30 : 54.1%	Primary : 32.4 %	Unemployed : 96.2%	Rural : 27.7%		
					≥30 : 20.6%	Tertiary : 56.8 %				
						Universal : 9.5%				
4	(Al-Riyami et al. 2011)	Oman	139	descriptive cross-sectional study	28 ± 5	No schooling: 2.2%;	Employed: 36%	NR	NR	First: 7.2%
					Range: 19–45	primary:15.8%;	Unemployed: 64%			Second: 36.0%
						secondary: 44.6%;				Third: 56.8%
						college:17.3%;				
						higher education: 20.1%				
5	(Ali-Shtayeh et al.^2015^)	Palestine	372	a cross-sectional survey	20_ 30 : 22.85%	Illiterate : 11.6%	Yes : 31.5%	City : 38.2 %	NR	NR
					31_ 40: 25.5 %	Primary: 26.6 %	No : 68.5%	Village : 53.5 %		
					More than 40 : 31.5 %	Secondary : 23.9 %		Camp : 4.3 %		
					Missing : 20.2%	University : 33.1 %		Missing : 4.0%		
						Missing : 4.8%				
6	(Al Essa et al. 2019)	Saudi arabia	297	A cross-sectional study	31.5 ± 6.0 years	Hight School : 69.36%	NR	Riyadh : 44.10%	First child	First : 14.14 %
						Lower education level : 16.83 %		Other : 46.46%	Yes : 20.53%	Second : 17.17 %
						Missing : 13.80		Missing: 9.42 %	NO :75.75 %	Third : 53.87%
									Missing: 3.70 %	Missing : 14.81
			
7	(Aljofan and Alkhamaiseh, 2020)	Saudi Arabia	879	Cross-sectional Study	8-29 : 49 %	None : 4 %	Housewife : 51 %	Urban : 62 %	NR	Currently pregnant : 71 %
					30-39 : 33 %	Hight School diploma : 52 %	Working full time : 49 %	Rural : 38 %		Previously pregnant : 29 %
					40- 49: 15 %	College : 43 %				
					>50 : 2 %					
					Mean ± SD. 29,5 ±n6,6 (18-53)					
8	(Abd El-Mawla 2020)	Egypt	400	A cross-sectional, qualitative	NR	Illiterate 4.25%	NR	Assiut 99.25 %	NR	NR
						Primary 7.00 %		Out Assiut 0.75 %		
						Intermediate 10.00 %				
						Higher School 29.25 %				
						University 49.50 %				
9	(Al-Ghamdi et al. 2017)	Saudi Arabia	612	A cross-sectional descriptive study	NR	No formal education : 4.4 %	Housewife: 49.2%	Saudi 84.40%	One : 19 %	NR
						Primary education : 5.4%	Private sector employee :7.8%	Non-Saudi 15.60%	Two : 17.6%	
						Secondary education : 8.5 %	Gouvemment employee: 39.7 %		Three: 18.6%	
						Hight school : 24.2%	Other : 3.3%		Four or more : 44.8 %	
						Diploma 13.4%				
						Bachelor’s degree : 41.2%				
						Master’s degree and higher : 2.9 %				
10	(Eid et al. 2020)	Palestine	350	cross-sectional observational study	Under 20 : 3.4%	Primarry and illeterate : 2%	Yes : 40 %	City : 39.1%	First child 29.5%	NR
					20-30 : 48.3%	Middle school : 15.7%	No (Housewife) : 60 %	Village : 59.7 %	Second 22.3%	
					31-40 : 29.4%	Hight School : 23.4%		Refuge camp : 1,2%	Third 21.4%	
					>40 years : 18.9 %	Graduate : 46.9 %			Fourth 32 9.1%	
						Postgraduate : 12%			Fifth 8.6 %	
									>5 9.1%	
11	(Sattari et al. 2012)	Iran	400	cross-sectional	15_19 : 5%	Hight school or lower 46 %	NR	City : 72.3%	None :45.8 5	NR
					20-24 : 31.8%	Diploma : 36.8 %		Village ; 27.8%	One : 29.5 %	
					25-29 :40 %	University education : 17.3 %			Two : 17.3 %	
					30-34 : 15.3 %				More : 7.5 %	
					35-39 : 7.5 %					
					40-44 :0.5 %					
					26.4 (±5.2) years					
12	(Abdollahi et al. 2019)	Iran	320	cross-sectional descriptive study	25-30 years of age with a mean of 27.97 ± 5.24 years	More than half of participants had an upper secondary level education (52.2%) as did about half of their husbands (47.6%).	Most of the women were housewives (91.2%)	NR	NR	NR
13	(Quzmar et al. 2021)	Palestine	400	a cross-sectional, non-interventional, descriptive study	Less than 25 : 26.3%		Housewife 81.5%	City : 37.0%	Pregnant (0) : 11%	NR
					25–34 : 42.0%	Elementary 4%	Governmental employee 11.3%	Village : 56.3 %	1–3 : 53.3%	
					More than 34 : 31.8%	Middle school 15%	Private sector employee 7.2%	Palestinian refugee camps : 6.82%	4-6 : 29.5%	
						High school 30.3%			>6 : 6.2%	
						University 50.8%				
14	(Alshehri 2021)	Saudi Arabia	340	a cross-sectional study	29.9 ± 5.6 years.	Illiterate/High school or less 37.10%	Housewife 67.60%	NR	1 : 31.70%	36 to 39 Week 51.50%
						Bachelor’s degree 55.90%	Teacher 15.30%		2 : 24.90%	40 Week 25.00%
						Postgraduate 7.10%	Health care provider 6.50%		3 : 18.90%	41 to 42 Week 23.50%
							Administrative 10.60%		4 : 9.80%	
									≥ 5 : 14.70%	
15	(Dabaghian et al. 2012)	Iran	600	Cross selectional study	27.03 ± 4.8	Uneducated: 2.5%;	Homemaker: 87.2%	Urban: 81.7%	1.6(0.81)	NR
						<12 years: 23.3%;	Others 12.8%	Rural:18.3%		
						12 years: 51. 8%;				
						>12 years: 22.4%				
16	(Hwang et al. 2016)	Iraq	335	a cross-sectional survey	20 years and bellow : 27.16 %	No schooling ; 22.38 %	Yes : 10.74%	Urban : 59.77 %	NR	NR
					21-30 years : 47.46 %	Elementary school : 35.52 %	No housewife : 89.25 %	Rural : 40.29 %		
					31-41 years : 25-37	Middle school or higher : 42.08 %				
					26.1 ± 6.9					
17	(Yazdi et al. 2019)	Iran	170	cross-sectional, multi-center study	26.6 (26.6 ± 5.67)	Illiterate 20.3%	House keeper 92.94 %	Urban 52.94 % Rural 47.05 %	Para parity mean (SD)	NR
						Under diploma 50.6%	Others 7.05 %		TM/CAM user 1.01 (1.06%)	
						Diploma 19.0%			TM/CAM non user 1.06 (1.11%)	
						University 10.1%				
18	(TableaTableaee 2011)	Iran	500	cross-sectional	≤25 (56.3%)	≤8 years: 63.5%	Homemaker: 94.3%	Urban: 29.6%	Primipara: 58.5%	NR
					26–30 (30.0%)	9–12 years: 25.1%		Rural:70.4%	Multipara:41.5%	
					31–35 (9.7%)	>12 years: 11.3%				
					>36 (3.9%)					
19	(Orief et al. 2014)	Egypt	300	cross sectional study	17–45	Illiterate 4%	NR	NR	min–max 0–7	First: 33.3%
					Mean ± SD 26.9 ± 4.9	Basic education 19%			Mean ± SD 1.2 ± 1.0	Second: 33.3%
						Secondary education or higher 77%				Third: 33.3%
20	(Al-Ramahi et al.^2013^)	Palestine	300	cross sectional study	Less than 20 7.33 %	Primary and illiterate : 6.6%	Yes : 5.66 %	City : 23%	First child : 32 %	NR
					20–30 : 69.66 %	Middle school :17%	No : 94.33 %	Village : 69.33 %	More than one : 68 %	
					31–40 : 21.66%	High school :37.33 %		Camp : 10 %		
					More than 40 : 1.33 %	Diploma/University education :39%				
21	(Saber et al. 2019)	Iran	150	cross-sectional study	17-41years of age with a mean of 26.2 ± 0.4 years	<12 years : 13.3%	Employed : 7.3%	NR		First:55.3%
						12 years : 61.3%	Housewife : 92.7 %			Second: 49.3%
						12-16 years : 22.7%				Third:41.3%
						>18 years :2.7%				
22	(Afshary et al. 2015)	Iran	801	descriptive-analytical study	Less than 25 years : 39.02 %	Diploma : (32.24%)	housekeeper : (71%)	NR	NR	NR
					25-30 years : 31.17 %	Being Educated in University: (37.56%)	Employed : 29 %			
					More than 30 years : 29.80 %					
23	(Aghababaei et al. 2019)	Iran	160	descriptive study	16-19: 15 %	Primary school : 8.8 %	House keeper : 87.5 %	NR	0 : 51.3 %	NR
					20-23 : 19.4 %	Middle school : 32.5 %	employee : 12.5 %		1 : 37.5 %	
					24-28 : 24.4 %	High school :41.8 %			2 : 6.2 %	
					29-32 : 20 %	college : 16.9 %			≥3 : 5 %	
					33-36 : 16.2 %					
					> 36 : 5 %					
24	(Raoufinejad et al. 2020)	Iran	400	single-centered, observational, retro-spective, cohort study	18 – 24 : 33.2 %	Under degree:34.8%	Unemployed: 91.7 %.	Urban : 74,5%	1: 64 %	1: 69.8 %.
					25 – 34 : 57.5%	Degree: 48.9%	Employed298 : 8.3 %	RuraL : 25.5%	≥ 2: 36 %	1 – 5 : 23.1%>
					35 – 44 : 9.2%	University : 16.3%				5: 0.9%
25	(Sadeghi & Mahmoud 2014)	Iran	420	a cross-sectional study	Lowest than 25 (26.2%)	Knowing reading and writing (7.1%)	NR	NR	NR	NR
					25-35 (23.8%)	Primary (11.9%)				
					35-45 (16.7%)	Secondary School level (16.7%)				
					45-55 (16.7%)	Technical Diploma (19.0%)				
					55 through highest (16.7%)	Intermediate (9.5%) +				
						Bachelor and masters degree (4.8%)				
26	(Khadivzadeh and Ghabel 2012)	Iran	919	cross-sectional study	Lowest than 18: (5.31%)	illiterate: 2.65 %	Unemployed: 95.81 %.	Urban : 13.68 %	NR	NR
					19-36 : (89.07%)	Elementary school : 24.61 %.	Employed : 0.81 %	RuraL : 86 %		
					> 36 : (4.90 %)	Hight school and diplomat: 66.18 %	companu employed and public officer : 2.65%	Missing: 0.3 %		
					missing : (0.71%)	complet academic studies : 5.92 % missing : 0.61 %	missing : 0.71 %			
27	(Amasha and Jarrah 2012)	Jordan	332	A descriptive cross- sectional study	<20 : 4.9 %	Less than secondary school : 25.7 %	Employed : 26.5 %	NR	NR	1 st Trimester: 15.5 %
					20-30. : 64.9 %	Secondary school : 50.2 %	Unemployed : 73.5 %			2nd Trimester :33.3 %
					31-40 : 27.8 %	Above secondary school (university/ college): 24.1%				3rd Trimester :40.3 %
					>41: 2.4 %					

### Risk of bias in studies

The findings of the quality appraisal of eligible studies were reported in Figure 1. The tool is used to indicate the methodological quality and appropriateness of the observational studies, including cross-sectional studies that were reviewed in this study. It consists of eight items, and we determined the score by counting the asterisks (*) that we gave to each answer to the eight items in the grids, where a high score indicates a higher quality of study and vice versa. The six of the 33 studies were evaluated by the abstract since these articles are in Persian language and we have received no response from their authors to retrieve the full text. Two reviewers completed this process, and where there were discrepancies, a team of reviewers intervened to resolve them.

### Results of syntheses

#### Sociodemographic characteristics of HM users

There was an important association between socio-demographic and obstetric characteristics of women and herbal medicine use. Most women in the included studies were from rural areas, homemakers, and had an educational qualification below graduation (Saadia et al. 2013; Afshary et al. 2015; Hwang et al. 2016; Botyar et al. 2018; Abdollahi et al. 2018, 2019; Yazdi et al. 2019; Raoufinejad et al. 2020). Most women in the included studies were from rural areas, homemakers, and had an educational qualification below graduation. However, a study from Saudi Arabia and Iran reported that women with a high school diploma or higher (i.e., those with at least 12 years of formal education) and women who were working full-time were significantly more likely to use herbal medicines during pregnancy compared to their less educated and unemployed counterparts (Aljofan & Alkhamaiseh 2020; Raoufinejad et al. 2020). In some studies, they found a significant relationship between age and the use of herbal medicines, with subjects aged between 20 and 29 years reporting the highest use of herbal medicines (Sattari et al. 2012; Orief et al. 2014; Botyar et al. 2018; Quzmar et al. 2021). The number of pregnancies and children also had a significant relationship with herbal medicine use, as women in their first pregnancy were mostly nonusers (Sattari et al. 2012; Quzmar et al. 2021). Two studies mentioned the association between ethnicities and herbal use (Botyar et al. 2018; Yazdi et al. 2019).

#### Most frequently used herbal medicines:

Table 2 shows that more than half of the studies listed the types of herbal medicines used by pregnant women, whereas the other half failed to indicate the kinds of HMs used by pregnant women (Table 2). Overall, 23 studies identified a total of 67 different medicinal plant species used in the traditional treatment of gestational health ailments/symptom complexes throughout EMRO’s region. Some studies have mentioned the use of mixed herbs by pregnant women without indicating the composition of these mixtures (Al-Ramahi et al. 2013; Saadia et al. 2013; Sadeghia and Mahmood 2014; Hwang et al. 2016).

**Table 2.** Prevalence and pattern of herbal medicine use, referral, and sources of information among pregnant women from the EMRO’s region.

N°	Study	Prevalance of use	Herbs used	Reason for use	Source of information	Trimester
1	Botyar et al. 2018	34.8%	NR	NR	from friends and family first and from books, magazines, the Internet, and medicinal plant shops’ clerks next.	NR
2	Saadia et al. 2013	66%	*Zingiber officinale* Roscoe 6.5% *Trigonella foenum-graecum* L. 5.40 % *Nigella sativa* L. 8.20% Take a combinaison 65.90% Nothing 8.20 %	NR	NR	NR
3	Biazar et al. 2019	19,6 %	*Cinnamomum verum* J.Presl : 15.4% *Mentha spicata* L. : 14.9% Khakshir Flixweld (Iranian syrups.):13.1% *Alhagi pseudalhagi* : 9.4% *Cichorium intybus* L. : 9.4 % *Primula veris* L. : 6.3 % *Zingiber officinale* Roscoe :5 % *Bunium persicum (*Boiss*.*) B. Fedtsch : 4.5% *Thymus vulgaris* L. :4 % *Camellia sinensis* (L.) Kuntze : 3.2% *Boswellia serrata* Roxb. ex Colebr : 2.7% *Matricaria chamomilla* L. : 2.7 % *Origanum vulgare* L : 2.7 % *Ziziphus mauritiana*1.8% *Ocimum americanum* L. : 1.4 % : 1.8% *Elettaria cardamomum* (L.) Maton: 1.4% *Crocus sativus* L.: 1.4 %	Reducing Blood Sugar/For Male Gender. GI Problems/UTI Common Cold Preventing Neonatal Jaundice Relaxation Nausea &Vomiting Relaxation/Common Cold GI Problems Weight Loss Intelligent Baby Muscle Pain Beautiful Baby	NR	NR
4	Al-Riyami et al. 2011	23,80%	*Zingiber officinale* Roscoe : 7.9% honey: 6.5% *Thymus vulgaris* L. : 5.0%; *Camellia sinensis* (L.) Kuntze : 3.6%	Flu and cold: 9.4%; flatulence: 6.5% Nausea: 5.0%; infection: 4.3%	NR	First:17.3% Second:17.1% Third:15.0%
5	Ali-Shtayeh et al. 2015	88.4%	*Salvia fruticosa Mill.* *Matricaria aurea* (Loefl.) Sch.Bip. *Pimpinella acuminata* (Edgew.) C.B. Clarke *Mentha spicata* L. *Cuminum cyminum* L.	abdominal pain, constipation, cold and flu, and flatulence	NR	NR
6	Al Essa et al. 2019	55.6%	Topical or oral olive oil (25.5%) *Cuminum cyminum* L.(19.5%)*,* *Allium sativum* L. (15.4%), *Zingiber officinale* Roscoe (13.4%), cranberry juice (13.4%), *Buninum persicum*(Boiss.)B.Fedtsch (11.6%) *Prunus mume (10.4%),* *Cinnamomum verum* J.Presl (10.4%)*,* *Trigonella* *foenum-graecum* L. (7.7%), and *Matricaria* *chamomilla* L. (4.3%)*.*	NR	37% of the respondents used herbs by their own initiative. 33% and 12% used herbs based on recommendations from their families and friends, respectively.	First : 50 % Second : 41.2 % Third : 60 %
7	Aljofan and Alkhamaiseh 2020	33%	NR	to improve the course of the pregnancy (56%) and to facilitate labour (49%)	NR	The first trimester (20%) The second trimester (45%) . The third trimester (35%)
8	Abd El-Mawla 2020	66.00 %	*Zingiber officinale* Roscoe *Mentha spicata* L. *Psidium guajava* L. *Matricaria chamomilla* L., *Echinacea purpurea* (L.) Moench, *Avena sativa* L., *Thymus vulgari*s L., *Cuminum cyminum* L. *Frankincense olibanum*	colds, cough, colic, nausea, vomiting, constipation, heart burn, dyspepsia, insomnia anxiety during pregnancy	NR	NR
9	Al-Ghamdi et al. 2017	25.3%	NR	NR	Myself 14.7% Herbalist 17.6% Family /freinds/relatives 52.9% Recommanded by medical doctor 10.3% Others 4.4 %	NR
10	Eid et al. 2020	77.1 %	*Mentha × piperita* L.*62%* *Salvia fruticosa* Mill. 58.6% *Pimpinella acuminata* (Edgew.) C.B. Clarke *50 %* *Camellia sinensis* (L.) Kuntze *Anthemis arvensis L* 47.4% *Matricaria chamomilla* L. 45.7% *Prunus dulcis* (Mill.) D.A.Webb 46% *Petroselinum crispum* (Mill.) Fuss *36%* *Coffea arabica* L. 29.4% *Allium sativum* L.19.1 % *Cinnamomum verum* J.Presl 17.4% *Rosmarinus eriocalyx* Jord. & Fourr. *17.1%* *Senna alexandrina* Mill. 4.6% *Zingiber officinale* Roscoe 8.0%	Flatulence Cramp Sleep aid, Expectorant, Cold (flu) Sleep aid Heartburn UT inflammation Stimulant Hypertension Inflammation Delivery aid Laxative	Myself 7.4 % Family : 25.1 % Friends : 50.3 % Doctor : 4 % Pharmasist : 6 % Media and internet : 6.3 %	NR
11	Sattari et al. 2012	22.3%	NR	NR	the subjects’ physicians (46.1%) or family members/friends (9%). Additionally, 44.9% reported self-medicated herbal medicine use.	NR
12	Abdollahi et al. 2019	46.4%	*Citrus × aurantium L.*(30.97%), *Mentha × piperita* L. (19.81%) *Borago officinalis* L. (19.46%) *Linaria vulgaris* Mill. (12.38%) and *Thymus vulgaris* L. (7.07).	to promote fetal health and intelligence (35.40%), to promote the women’s health status (32.5%), to relieve discomforts during pregnancy (25.1%), to restore youth (3.6%) to facilitate labor, abortion, reproductive health (16.1%)	friends and relatives (30.5%), their mothers (26.3%) mass media (11%)	first (10.49%) second trimester of pregnancy (6.26%) third (25.96%),
13	Y Quzmar et al. 2021	25.3%	*Salvia officinalis* L. *: 10.8%* *Thymus vulgaris* L.*: 8.3%* *Mentha × piperita* L. *: 11.5 %* *Trigonella foenum*-*graecum* L. *: 5.3 %* *Matricaria chamomilla* L. *: 9.5 %* *Pimpinella acuminata* (Edgew.) C.B. Clarke *: 12.3 %* *Zingiber officinale* Roscoe *: 5.3%* *Cuminum cyminum* L.*: 7%* *Cinnamomum verum* J.Presl *: 9%* *Curcuma longa* L. *: 2 %* *Allium sativum* L. *: 4.5 %* *Petroselinum crispum* (Mill.) Fuss *:6.8%* *Camellia sinensis* (L.) Kuntze*:2.8%* *Crocus sativus : 1.5 %* *Rosmarinus officinalis* L.*: 1.8%* *Panax ginseng* C.A.Mey. *: 0.5 %* *Oleaceae Olea europaea : 6.8%* *Ricinus communis* L.*: 4.5 %* *Vitis vinifera* L.*seed oil): 0.3 %* *Almond oil (Prunus amygdalus): 0.5 %* *Aloe perfoliata* L.*: 0.8 %* *Vaccinium macrocarpon* *Aiton* : 1.3 %	NR	Physician : 13.5 % Midwife , nurse : 4.5 % Pharmacist : 2.3 % Television : 11.5 % Radio : 5.5 % Family : 24.3 % Neighbours : 18.3 % Social media : 17.3 %	NR
14	Alshehri 2021	31.50%	NR	to make childbirth easier .	The most common sources of herb supply were relatives for over half of the participants (53.8%). self-sourced (29.8%). social media (21.2%). based their decision 13.5%. the participants asked their doctors and a smaller(9.6%). Pharmacist (3.8%)	36 to 39 Week 18.30 % 40 Week 32.90 % 41 to 42 Week 58.80%
15	Dabaghian et al. 2012	67.0%	*Mentha × piperita* L.: 32.8% *olibanum:* 26.3%	Bloating, stomach ache:30.2%; respiratory infections:18.7%nausea; vomiting:11.5% heart burn:8.7% anxiety:4.7%, Sleep disorders:4.7% skin problems:2.9%	Family, friends: 60.2% Books/newspapers/magazines/internet: 49.7% Herbal store/ pharmacy: 26.7% Television: 15.5% Physician: 12.8%	NR
16	Hwang et al. 2016	56.7 %	*Nigella sativa* L. : 16.5% *Matricaria chamomilla* L. : 16.2% *Cinnamomum verum* J.Presl : 10.8% *Ricinus communis* L. *:* 9.4 % *Zingiber officinale* Roscoe : 8.3% Royal honey : 6% *Allium sativum* L.*:* 5.4 % *Mentha × piperita* L.*:* 4 % *Olea europaea* L. *:* 3.1 % Unidentified mixed spices : 2.8% *Cuminum cyminum* L.*:* 2.3 % *Glycyrrhiza glabra* L. *:* 2.3 % *Thymus vulgaris* L. : 1,1 % *Eucalyptus globulus* Labill. 0.9 % Camellia sinensis* (L.)* Kuntze*:* 0.9 % Special type of spices : 0.6 %	NR	Friends : 46.3 % Family : 14.8 % Neighbor : 16.3 % Newspaper : 16.3 % Television ,radio :6.3 % Religious group :1.8% Health professionals : 1.1%	NR
17	Yazdi et al. 2019	89.9%	herbal medicine 93.0% *Cichorium intybus L.* *and* *Salix caprea* L.*)* dry herbal water vapor 6.3% an animal-derived drugs 1.3%	for the prevention of jaundice in newborns.	89.9% of the women used TM/CAM based on the recommendation of friends and family .	the third trimester
18	TableaTableaee 2011	82.3%	*Ammi majus L*. : 22.6% *Thymus vulgaris L*. : 12.6% *Ocimum basilicum L*. : 28.0% *Mentha × piperita L*. : 9.0% *Zingiber officinale* Roscoe : 5.4% *Cinnamomum verum* J.Presl : 5.4% *Matricaria chamomilla* L. : 4.5%	GI related problem: 32.1%nausea and vomiting:20.2% Urinary tract infection:5.5% sedation: 4.6% increase child intelligence: 3.7% prevent neonatal hyperbilirubinemia:14.7% common cold:11.0%	Family: 87.3% primary maternity care providers: 7.6% friends; neighbors: 2.5% midwife: 2.5%	First: 36.7% Second:15.2% Third: 31.0% Any time: 17.1%
19	Orief et al. 2014	27.3%	*Zingiber officinale* Roscoe 29.3 % *Mentha × piperita* L. : 11 % *Trigonella foenum-graecum* L. 31.7 % *Pimpinella anisum* L : 40.2% *Camellia sinensis (L.)* Kuntze: 19.5% *Allium sativum* L.*: 22%*	Nausea and vomiting 28 %Abdominal colic 47.6% Dysuria 9.7 % Headache 2.4% No indication 18.2%	Physicians 11 % Female herself 18.3 % Friends 7.5 % Family 42.7 %	1st Trimester 25.6 % 2nd Trimester 36.6 % 3rd Trimester 37.8 %
20	Al-Ramahi et al. 2013	40.0%	*Pimpinella anisum* L *:* 61.7% *Matricaria chamomilla* L. 53.3% , *Salvia officinalis L.* 45.8* % ,* *Allium sativum* L. *:* 2.5 %. *Caraway* 2.5 % *Mentha × piperita* L. 14.2%. *Cinnamomum verum* J.Presl 10.8 %. *Trigonella foenum*-*graecum* L. 9.2 %. *Cuminum* *cyminum* L. 6.7 %. *Zingiber officinale* Roscoe 3.3 % *Foeniculum vulgare* Mill. 1.7 % *Prunus dulcis* (Mill.) D.A.Webb 1.7%. *Petroselinum crispum* (Mill.) Fuss 1.7%. *Ricinus communis* L. : 0.8 %. *Camellia sinensis (L.) Kuntze:* 0.8 % *Syzygium aromaticum* (L.) Merr. & L.M.Perry *0.8 %.,* *Nigella sativa L.* 0.8 % *Citrus limon* (L.) Osbeck 0,8 %* ,* *Capparis spinosa* L. *0.8 %* Mixture of herbs 33.3%*,* *Thymus vulgaris* L. 29.2 % , Dates 28.3%	Flue and cough. Abdominal pain Vomiting Diuretic Chest pain Laxative Infections Flatulence Relaxation Stomachache, heartburn Teeth pain path Urinary tract infections AmebaApiaceae , Facilitate delivery	NR	NR
21	Saber et al. 2019	71.3%	*Mentha × piperita* L. : 30* %* *Borago officinalis* L : 19.3* %* *Edible frankincense* :* 18.7%* *Matricaria chamomilla* L. *Sisymbrium irio* L. *:* 14 %	Improving gastrointestinal 66.6%problems and reducing nausea Strengthening the nerves and sedation 26 % Increasing fetus’ IQ : 20 %	NR	Second: 10.4%, Third: 15.1% During whole pregnancy : 32.1%
22	Dabirifard et al. 2017	90.2%	herbal tea 73.64% *Zingiber officinale* Roscoe 41.08% *Mentha × piperita* L. 30.23%	to control nausea and vomiting to control heartburn	The information sources was family and friends: 94.57%	NR
23	Afshary et al. 2015	60%	NR	treatment of digestive disorders (50%), skin diseases (34.4 percent) and cold (15.6%) ; the medicinal plants most frequently used as a treatment of digestive disorders (66.9%), hypoglycemia (17.8%), treatment of anemia (5.1%) and treatment of renal disorder (5.1 %) and finally cold treatment (5.1 %).	friends and family (52.17%)	NR
24	Aghababaei et al. 2019	50%	NR	NR	NR	NR
25	Abdollahi et al. 2018	48.4%	*Citrus × aurantium* L. (30.76%) and *Mentha × piperit*a L. (2lokj 2%)	to promote fetal health and intelligence (28.3%).		
26	Hosseini et al. 2017	63.40%	NR			
27	Karimian et al. 2019	57.1%	NR			
28	Soleymani & Makvandi 2018b	19.2%	*Thymus vulgaris* L., *Olea europaea* L., and *Peganum harmala* L.	cold and cough		
29	Ekrasarian et al. 2017	48%	*Mentha × piperita* L. (96.7%) *Citrus × aurantium* L. (89.6%) Cinnamomum verum J.Presl (85.7%)*.* And *Descurainia sophia* (L.) *Webb ex Prantl* *(22%)*	NR	NR	NR
30	Raoufinejad et al. 2020	81.3%)	*Mentha × piperita* L. (47.4%)*.* Frankincense (44.3%) *Descurainia sophia* (L.) *Webb ex Prantl* (40.6%) *Olea europaea* L. *(39.4 %)* *Cinnamomum verum* J.Presl (20.3 %) *Borago officinalis* L (18.2%) *Thymus vulgaris* L. (16.9%) *Mentha pulegium* L. 47 (14.5) C*ichorium intybus* L. (13.2%). *Zingiber officinale* Roscoe (12.3 %) *Crocus sativu*s L. (10.8%)	Treatment of flatulence, abdominal pain, heartburn, nausea/vomiting, and indigestion . Improving fetus brain development. Reducing the risk of neonatal jaundice; treatment of consti-pation. Treatment of stretch marks, and constipation Treatment of hyperglycemia, heartburn, flatulence; habitual use. Treatment of palpitation, cold/flu; relaxation; delivery in-duction; habitual use Treatment of infections; habitual use. Reducing the risk of neonatal jaundice; habitual use. Treatment of nausea/vomiting, heartburn, flatulence; habit-ual use	93.0% preparations were provided from places other than drug-stores. 14.5% were recommended by healthcare providers. 49.1% of uses were advised by family/friends. 29.6%. were based on personal experi-ences.	NR
31	Sadeghi and Mahmood 2014	36%	*Pistacia aethiopica* Kokwaro, *Carum carvi* L., *Foeniculum vulgare* Mill., *Anethum foeniculoides* Maire & Wilczek, *Trigonella foenum*-*graecum* L. *Crocus sativus* L. *and* *Nigella sativa* L., *Quercus infectoria* G.Olivier, *Piper nigrum* L. and *Zingiber officinale* Roscoe	Abortion: 22.14 % Aphrodisiac: 57.85 % Anemia : 68.33 % Contraception management 31.90% Digestive problem: 67.14 % Gestational diabete: 53.09 % Gestational hypertension and hyperlipidemia: 54.76% Infections: 54.76 %	NR	NR
32	Khadivzadeh and Ghabel 2012	49.2 %	NR	gastro intestinal problems, low blood pressure and urticarial	friend, colleague and family members 33.5%; obstetricians and midwives 41.5%; books, journals and magazines 6.7%; traditional healers 3.6%; radio and television 5.1%, internet. 3% and unknown 9.3%.	NR
33	Amasha and Jarrah 2012	73.79%	*Pimpinella anisum L31.4%),Salvia officinalis* L. (23.3%). *Zingiber officinale Roscoe* 3.7%* .* *Mentha *×* piperita* L. 14.3 %* ,* *Thymus vulgaris* L. 4.1 %, *Senna alexandrina* Mill. *Cinnamomum verum* J.Presl 4.1% ,	Relax / Help sleep / Colic Heart burn / Vomiting Constipation / pills Nausea / Vomiting Epigastric pain Common cold / Bronchitis Nausea / Vomiting Facilitate labor Chronic Headache / Back ache Vaginitis / Piles cramp / skin itching / muscle body ache Back pain Piles	My own idea / Self preference: 15.1 %. Mother/ Mother-in-low : 61.6 % Friends : (5.7%) Obstetricians and nurses: (9.0%) Others : (6.5%) Given more than one source: (2.1 %)	NR

The most commonly used herbs identified were: ginger *Zingiber officinale* Roscoe (Zingiberaceae), thyme *Thymus vulgaris* L. (Lamiaceae), peppermint *Mentha × piperita* L. (Lamiaceae), sage *Salvia officinalis* L. (Lamiaceae), chamomile *Matricaria chamomilla* L. (Asteraceae), fenugreek *Trigonella foenum-graecum* L. (Leguminosae), Black seeds *Nigella sativa* L. (Ranunculaceae), honey, cinnamon *Cinnamomum verum* J.Presl (Lauraceae), sour orange blossom *Citrus × aurantium* L. (Rutaceae), green tea *Camellia sinensis* (Theaceae), aniseeds *Pimpinella anisum* L. (Apiaceae), garlic *Allium sativum* L. (Amaryllidaceae) and cumin *Cuminum cyminum* L. (Apiaceae). Various studies reported the parts of the plant used, methods of preparation, and routes of administration. Most of the users customarily use diverse plant parts as medicinal agents, including root, bark, fruit, bulbs, whole plants, rhizomes, seeds, flowers, and stems, but leaves were the predominant part used (Sadeghia and Mahmood 2014; Ali-Shtayeh et al. 2015; Eid et al. 2020). Some of the studies explored the method of preparation of medicinal herbs for treatment. We have observed that the most common methods were infusions/tea, maceration, squeezing, chewing, decoction, bathing, evaporating/inhaling, and ingestion of raw medicinal plant material (Sadeghi and Mahmood 2014; Ali-Shtayeh et al. 2015; Eid et al. 2020). Routes of administration included oral (Al-Ramahi et al. 2013, Ali-Shtayeh et al. 2015, Raoufinejad et al. 2020), topical (Sadeghi and Mahmood 2014; Ali-Shtayeh et al. 2015; Raoufinejad et al. 2020) and intra-vaginal (Al-Ramahi et al. 2013). Furthermore, oral ingestion was the most common method and was suggested in numerous studies. In some studies, they found a significant relationship between the trimester of pregnancy and the use of herbal medicines (Saadia et al. 2013; Afshary et al. 2015; Yazdi et al. 2019; Al Essa et al. 2019; Alshehri and Alshehri 2021). The majority of the studies reported the highest use of herbs during the third trimester, with the frequency varying from 15.3% to 60.0% (Al-Riyami et al. 2011; Tabatabaee 2011; Orief et al.^2014^; Abdollahi et al. 2019; Al Essa et al. 2019; Saber et al. 2019; Yazdi et al. 2019; Aljofan and Alkhamaiseh 2020; Alshehri and Alshehri 2021).

#### Reasons for use and sources of information

Users of herbal medicines during pregnancy had several reasons for consuming these medicines. Informants in most of the studies reported the use of herbal medicines to alleviate pregnancy-associated symptoms. The herbs were most frequently used to treat gastrointestinal disorders such as nausea, vomiting, abdominal pain, bloating, flatulence, and stomach aches, followed by cold and flu symptoms and stretch marks. Although some others reported this use for stimulation of labor, and facilitation of childbirth. Other uses were specifically to enhance neonates intelligence and promote fetal health. Finally, skin problems, sleep disorders, and weight loss are the lesser common reasons that we have noted among some users. Finally, reported traditional indications of the most frequently used herbal medicines are shown in (Table 2). The quality and source of information received on herbal medicine automatically influence the choice of treatment for maternal illnesses. Nearly half of the reviewed materials disclosed the sources from which pregnant women received information about herbal medicines. The principal sources were family and friends respectively, for the studies (TableaTableaee 2011; Hashem Dabaghian et al. 2012; Amasha and Jarrah 2012; Khadivzadeh and Ghabel 2012; Hwang et al. 2016; Al-Ghamdi et al. 2017; Abdollahi et al. 2019; Al Essa et al. 2019; Yazdi et al. 2019; Eid et al. 2020; Raoufinejad et al. 2020; Quzmar et al. 2021). Some women decided in-person to use herbal medicines in pregnancy, labor, or postpartum (Sattari et al. 2012; Al-Ghamdi et al. 2017; Al Essa et al. 2019; Eid et al. 2020; Raoufinejad et al. 2020; Alshehri and Alshehri 2021). On the other hand, some expectant women indicated health professionals such as physicians, pharmacists, and nurses as a source of herbal medicine information (Amasha and Jarrah 2012; Hashem Dabaghian et al. 2012; Orief et al. 2014; Hwang et al. 2016; Eid et al. 2020; Raoufinejad et al. 2020; Quzmar et al. 2021; Alshehri and Alshehri 2021). Other users qbtainedtheir recommendations from the bush, traditional herbalists, the internet, social media, newspapers, radio, television, and books.

#### The motivation for HM utilization

The time required to decide to use HM varied greatly depending on their health needs, current knowledge, and habits. According to some studies, women’s personal preferences influenced their choices and decisions. A study from Iran (Abdollahi et al. 2018) reported women’s perception of the applied herb’s efficacy, and 41.2% of users were fully satisfied. Women who used HM before and during gestation seemed more probable to use it throughout labor and after delivery as for example, in a study from Saudi Arabia (Afshary et al. 2015). The primary motivations to utilize herbal medicines or herbal remedies during pregnancy are that they had been taken before the pregnancy and to save money on healthcare costs. Others believe they are safe and not dangerous herbs for pregnant women and embryos (Amasha and Jarrah 2012; Khadivzadeh and Ghabel 2012; Sattari et al. 2012). Moreover, some women made decisions based on their health conditions, and according to their experiences. In Palestine, 21.0% of the participants reported that they used HM because medical therapies failed to succeed. Nearly 58% used HM because it was more accessible, compared to medical therapy. Other participants used CAM because of its common use and recommendation in their culture (Quzmar et al. 2021).

## Discussion

To our knowledge, our work represents the first systematic review to identify the prevalence, motivation, and factors associated with herbal use among pregnant women in EMRO countries. This current review included 23 cross-sectional studies that included data on 13,021 women involved in the respective studies.

The findings suggest that the prevalence of herbal medicine use among pregnant women from the EMRO countries varied from 19.2% to 90.2%. This finding broadly supports the work of other studies in this area, such as a recently published systematic review by Ahmed et al. (2018) of 50 studies, including a total of 22404 African pregnant or lactating women, showing that the average prevalence of herbal medicine use during pregnancy among the different African regions was between 32% (in Central Africa) and 45% (in East Africa) (Ahmed et al. 2018). Additionally, current studies have highlighted the difference in the prevalence of herbal medicine among pregnant women, 59.3% in Yamen (Ahmed et al. 2022), 51.2%-65.6% in Ethiopia (Belayneh et al. 2022; Wake and Fitie 2022), 71.80% in Bangladesh (Jahan et al. 2022), and 67.45% in Morocco (Kamel et al. 2022). To date, many studies have investigated the impact of herbal medicine on pregnant women in another geographic area, a systematic review of Asian countries reported that, in total, 1283 out of 2729 (47.01%) women used at least one herbal medicine during their pregnancy (Ahmed et al. 2017). In Europe, North, and South America systematic review reported that, in total, 29.3% of the women (*n* = 2673) used herbal medicines during pregnancy (Kennedy et al. 2016). Based on the survey of the literature related to our topic, some research focuses on the prevalence of herbal medicine, such as a preliminary study of pregnant women, which reported a prevalence of 57.3% (Barnes et al. 2022), a further study found that the prevalence total was 65.71% of women that used Chinese herbal medicine formulas, including 26.13% during pregnancy and 55.63% after delivery (Xiong et al. 2023).

These findings suggest that herbal medicine use during pregnancy is not only common in EMRO’s countries as an entrenched part of the culture but is also common elsewhere in developed societies where traditions do not directly impact this use. Regarding the use of herbal medicine in pregnancy, most women in the studies were from rural areas, homemakers, and had an educational qualification below graduation. We found that there was a strong interplay of sociodemographic between the obstetric characteristics of women and herbal medicine use. This was in accordance with studies outside of EMRO countries that reported higher usage of herbs among women from rural areas that were less educated, these findings have been documented in previous studies in several countries around the world such as Kenya (Mothupi 2014), Bangladesh (Jahan et al. 2022), Uganda (Kaadaaga et al. 2014), and Ethiopia (Belayneh et al. 2022). In addition, it was observed that some of those women perceived that they had better knowledge about herbal medicine than the physicians/nurses and would persist in using it against medical advice. In contrast, a study in Norway and the United States shows a difference in the characteristics of herbal medicine users, the differences can be explained by the socioeconomic levels of families. The rich consult with their doctor before use or otherwise ensure adequate information about the benefits and side-effects of the herbal product (Nordeng and Havnen 2004; Bercaw et al. 2010).

The data of our systematic review contradict the widely-held hypothesis that uneducated housewives living in rural regions are the most probable users of high-risk, untested products, such as herbal or plant-based products. However, in addition to the current results, a previous study examining the prevalence of use and costs of herbal medicines in northern Scotland demonstrated that educated women were more likely to use this medicine (Pallivalapila et al. 2015). Similar findings are also reported in these studies (Mothupi 2014; Hwang et al. 2016). Based on WHO findings, two-thirds of women in low-middle-income countries (LMICs) use herbal medicines as their primary source of health care (Crockett et al. 2020). Some research studies (Botyar et al. 2018; Yazdi et al. 2019) reported the relationship between ethnicities and the use of herbal medicines, these studies indicated that the Arab population used traditional medicine more than the Fars population during pregnancy. However, there is a lack of data on the use of herbal medicines among different ethnic populations worldwide (Graham et al. 2005; Rashrash et al. 2017).

The current survey showed that pregnant women from EMRO’s region use a wide diversity of herbal medicine, and a total of 65 different medicinal plant species used in the traditional treatment of gestational health are reported. Generally, the African continent is known for its rich biodiversity. This species richness was reflected in the systematic review by Ahmed et al. (2018) in which the number of cited plant species varied from study to study, and 274 medicinal plants were reported to be used by African pregnant women. The following herbs were the most commonly used: (*Zingiber officinale*), (*Thymus vulgaris*), (*Mentha × piperita*), (*Salvia officinalis*), (*Matricaria chamomilla*), (*Trigonella foenum-graecum*), (*Nigella sativa*), honey, (*Cinnamomum verum*), (*Citrus × aurantium*), (*Camellia sinensis*), (*Pimpinella anisum*), (*Allium sativum*) and (*Cuminum cyminum*). The frequently used herbs in most studies were similar to other studies from the EMRO. In sub-Saharan Africa, the top herbal medicines cited in the studies were *Zingiber officinale*, *Allium sativum, Cucurbita pepo* (Cucurbitaceae), *Ricinus communis* (Euphorbiaceae)*, Vernonia amygdalina* Debile (Asteraceae) and *Garcinia kola* Heckel (Clusiaceae). *Zingiber officinale* was the most common species for the treatment of pregnancy-induced nausea and vomiting and was reported in 15 studies (El Hajj et al. 2020). In contrast, due to differences in culture, traditions, and climate, it is expected that herbal medicines used during pregnancy vary across countries and regions. The herbs most commonly used in Australia (Barnes et al. 2022), Norway (Nordeng and Havnen 2004), and Tuscany were *Rubus idaeus* L. (Rosaceae)*, Foeniculum vulgare,* and *Hypericum perforatum* L. (Hypericaceae). A good variety of reasons were proffered for using HM. The most common reason was to alleviate pregnancy-associated symptoms. The herbs were most frequently used to treat gastrointestinal disorders such as nausea, vomiting, abdominal pain, bloating, flatulence, and stomach aches, followed by cold and flu symptoms. Others reported this use for stimulation of labor or facilitation of labor and delivery. Other uses were specifically to enhance neonates’ intelligence and promote fetal health. Finally, skin problems, sleep disorders, and weight loss are a few reasons that we have noted among some users. The quality and source of information obtained on herbal medicine immediately impact the choice of therapy for maternal disorders.

Family and friends were the common referral/information sources in the studies (Tablea 2011; Hashem Dabaghian et al. 2012; Amasha and Jarrah 2012; Khadivzadeh and Ghabel 2012; Hwang et al. 2016; Al-Ghamdi et al. 2017; Abdollahi et al. 2019; Al Essa et al. 2019; Yazdi et al. 2019; Eid et al. 2020; Raoufinejad et al. 2020; Quzmar et al. 2021). Some women decide to use herbal medicines in-person during pregnancy, labor, or postpartum. Some pregnant women, on the other hand, mentioned health professionals such as physicians, pharmacists, and nurses as source of information on herbal medicines. To provide optimal care and counseling for patients who use herbal drugs, pharmacists need to be well-informed about the use and safety of herbs and should update their knowledge. This can be achieved by providing education and training to practicing pharmacists by organizing continuing medical education programs focused on the efficacy, potential risks, possible herb-drug interactions and consequences, and the key principles applied to the administration of herbs during pregnancy. We would also advise that physicians specifically ask about herb usage and document it in the patient record. Pregnant mothers should be informed of the potential risks posed by herbs during pregnancy and advised to avoid their use. Other users obtained their recommendations from the bush, or traditional herbalists, the internet, social media, newspapers, radio, television, and books. In these cases, it is necessary to exploit this point to carry out awareness and create preventive programs, including a media campaign that uses digital conversational advertisements broadcast on sites consulted by pregnant women or those planning to become pregnant, as well as banners and videos. The multiplicity of the points of contact will ensure the visibility of this type of message to the target audience, e.g., building on the community approach with pair training that will facilitate the dissemination of the message in the community.

The time taken to decide to use HM varied considerably depending on women’s health needs, available information, and preferences. Some studies discovered that women’s personal preferences motivated the choices and decisions they made. For most users, HM was the ideal medicine for solving pregnancy problems, inducing labor, and treating postpartum complications (Bercaw et al. 2010). Women who used HM during pregnancy and pre/pregnancy were more likely to use it during labor and after delivery. The most common reasons for using herbal medicines or herbal remedies during pregnancy are that they were using them before pregnancy, they had costly medical expenses, they are safe and harmless herbs for the mother and fetus, and they used HM because it was more accessible, compared to medical therapy. These findings were reported in other locations (Agyei-Baffour et al. 2017; Sumankuuro et al. 2017; Ahmed et al. 2022; Elba et al. 2022). Moreover, some women made decisions based on their health conditions, and their experiences. The participants used herbal medicine because of its common use and recommendation in their culture. These findings were reported in other studies (Tripathi et al. 2013).

### Limitations of the studies

This review contains some limitations, such as the fact that the prevalence identified may not represent the true prevalence due to the variations in the studies. Also, we have found that the majority of the published literature is predominantly from one country.

## Conclusions

Overall, our systematic review is one of the first reports to shed light on the prevalence, use pattern, and perceptions of herbal medicine use among pregnant women in the EMRO’s region. A low-disclosure study of herbal medicine use among pregnant women in Morocco has also been shown in the present review. Despite the importance of literature data about the use of herbal remedies, it is necessary to obtain good knowledge, attitude, and motivation for herbal consumption among women. Healthcare professionals and researchers can disseminate the results of this study to choose the best ways to hide the message in the context of prevention. Herbal medicines may be natural, but they do contain pharmacologically active ingredients. Due to the limited number of studies, it is recommended that future studies focus on safety and the effects of herbal medicines on pregnancy outcomes.
